# Causes of Childhood Cancer: A Review of Literature (2014–2021): Part 2—Pregnancy and Birth-Related Factors

**DOI:** 10.3390/cancers17152499

**Published:** 2025-07-29

**Authors:** Rebecca T. Emeny, Angela M. Ricci, Linda Titus, Alexandra Morgan, Pamela J. Bagley, Heather B. Blunt, Mary E. Butow, Jennifer A. Alford-Teaster, Raymond R. Walston III, Judy R. Rees

**Affiliations:** 1 Department of Internal Medicine, Division of Molecular Medicine, UNM Comprehensive Cancer Center, Cancer Control & Population Sciences Research Program, University of New Mexico Health Sciences, Albuquerque, NM 87131, USA; 2 Department of Pediatrics, Division of Pediatric Hematology-Oncology, Dartmouth Health Children’s, Lebanon, NH 03756, USA; 3 Department of Pediatrics, Geisel School of Medicine at Dartmouth, Dartmouth Cancer Center, Hanover, NH 03755, USA; 4 Department of Obstetrics and Gynecology, Dartmouth Health, Lebanon, NH 03756, USA; 5 Biomedical Libraries, Geisel School of Medicine at Dartmouth, Hanover, NH 03755, USA; 6 New Hampshire Department of Health and Human Services, Concord, NH 03302, USA; 7 New London Hospital Association, New London, NH 03257, USA; 8 Department of Pediatric Hematology Oncology, Children’s Hospital Colorado, Aurora, CO 80045, USA; 9 Department of Epidemiology, Geisel School of Medicine at Dartmouth, Dartmouth Cancer Center, Hanover, NH 03755, USA; 10 Public Health Institute, Cancer Registry of Greater California, Sacramento, CA 95825, USA

**Keywords:** pediatric cancer, epidemiology, etiology, tumor, perinatal, pregnancy

## Abstract

Purpose: To review parental pre-pregnancy and pregnancy exposures in relation to pediatric cancer (diagnosis before age 20). Methods: We conducted literature searches using Ovid Medline and Scopus to find primary research studies, review articles, and meta-analyses published from 2014 to 17 March 2021. Results: Strong evidence links increased risk of childhood cancer with maternal diabetes, age, and alcohol and coffee consumption during pregnancy. Both paternal and maternal cigarette smoking before and during pregnancy are associated with childhood cancers. Diethylstilbestrol (DES) exposure in utero has long been known to be causally associated with increased risk of vaginal/cervical cancers in adolescent girls. More recent evidence implicates in utero DES exposure to testicular cancer in young men and possible intergenerational effects on ovarian cancer in the granddaughters of women exposed to DES during pregnancy. There is strong evidence that childhood cancer risk is also associated with both high and very low birth weight and with gestational age. Evidence is also strong for the protective effects of maternal vitamin consumption and a healthy diet during pregnancy. Unlike early studies, those reviewed here show no association between in utero exposure to medical ionizing radiation, which may be explained by reductions over time in radiation doses, avoidance of radiation during pregnancy, and/or by inadequate statistical power to detect small increases in risk, rather than a lack of causal association. Evidence is mixed or conflicting for an association between childhood cancer and maternal obesity, birth order, cesarean/instrumental delivery, and prenatal exposure to diagnostic medical radiation. Evidence is weak or absent for associations between childhood cancer and multiple gestations or assisted reproductive therapies, as well as prenatal exposure to hormones other than DES, and medications.

## Simple Summary

Although the causes of childhood cancer have been widely investigated, the evidence in many areas is inconclusive. We searched studies published from 2014 through 2021, that investigated the etiology of childhood cancer. We have summarized our findings in three papers: risk factors during childhood (Part 1), parental and pregnancy-related factors (Part 2), and environmental and occupational risk factors (Part 3). In this paper, Part 2, we summarize evidence from studies assessing perinatal exposures and childhood cancer risk. We also identified evidence of inverse, protective associations of childhood cancer risk with dietary and vitamin intake during pregnancy. This synthesis of peer reviewed literature may help evidence-based education efforts to reduce the incidence of pediatric cancer.

## 1. Introduction

This is the second manuscript in a three-part series reviewing published studies of risk factors for childhood cancers. Here, we summarize evidence from studies published during the period January 2014–March 2021 that assessed parental exposures (Table 1) from before conception through the child’s birth.

## 2. Materials and Methods

The methods for this study, including the detailed search strategy, were described previously [1]. Briefly, we identified primary research studies and reviews in Ovid Medline and Scopus from 1 January 2014 through 17 March 2021. No restrictions were applied. Search strategies were adjusted for the syntax of each database. Methods for the systematic search of the literature are detailed in Ricci et al. 2024 [1] and the complete search strategy employed for each database is provided in the Supplementary Table S1 of Ricci et al. 2024 [1]. Inclusion criteria for primary research studies required subject headings and text words representing four concepts: childhood cancer, cause, exposure, and measures of association. Both general terms and specific cancer names listed by the International Classification of Childhood Cancer (3rd edition) were used to search childhood cancer [2]. For the exposure concept, general words such as ‘exposure’ or ‘environmental’ as well as specific potential cancer-causing agents or classes of agents were used, e.g., diethylstilbestrol, fertility treatment. For the cause concept we included words such as ‘risk’, ‘etiology’ or ‘cluster’. To find studies that focused on an association between childhood cancer and exposure, we included words such as ‘association’, ‘rate ratios’, or ‘relative risk’. To find review articles we searched for text words representing three concepts: childhood cancer, exposure and cause. Review articles were identified if they were tagged by the database as a review or included ‘review’, ‘systematic’, ‘metaanalysis’, ‘meta-analysis’, or ‘scoping’ in the title. We used a team-based approach to review 520 research articles and 462 reviews, summarizing the results by topic (Table 2), and discussing the weight of evidence for each. In this review we have synthesized published literature on the risk of childhood cancer associated with parental pre-pregnancy- and pregnancy-related factors. Except for large or influential studies, we generally omitted individual studies that contributed data to the meta- or pooled analyses reviewed here. Observed risk estimates are presented as odds ratios (ORs), adjusted OR (aOR), relative risk (RR), incidence rate ratio (IRR), adjusted IRR (aIRR), hazard ratio (HR), and excessive relative risk (ERR), with 95% confidence intervals (CIs).

## 3. Alcohol

IARC classifies alcohol as a group I carcinogen, ‘carcinogenic to humans’ [3]. There is strong evidence of a link between mothers’ alcohol consumption during pregnancy and elevated cancer risk in their children. A meta-analysis of 39 case–control studies including over 16,000 cases of childhood leukemia found a dose–response relationship between perinatal exposure to maternal alcohol consumption and AML; children born to mothers with high alcohol consumption were more than twice as likely to develop AML compared with children born to women with no alcohol consumption (OR 2.36; 1.60–3.49) [6]. A meta-analysis of six case–control studies found increased risk of neuroblastoma in children whose mothers drank any alcohol compared with no alcohol during pregnancy (OR 1.26; 1.07–1.49) [7]. A nationwide case–control study in Greece showed an increased risk of all CNS tumors in children born to women who drank alcohol between 3 months before conception through 3 months after delivery (aOR 2.35; 1.45–3.81) [8]. An association between drinking before pregnancy and elevated risk of Wilms tumor was shown by a meta-analysis combining the nation-wide case–control study in Greece and four other studies of mixed design (OR 5.31; 2.00–14.10) [9]. A similar magnitude of effect was observed for drinking before/during pregnancy, but the confidence interval was wide and included the null, indicating a nonsignificant statistical association (OR 5.30; 0.87–32.50). The previously mentioned meta-analysis by Karalexi also assessed paternal alcohol consumption but did not find a significant association with risk of childhood cancer [6].

## 4. Smoking

IARC classifies tobacco smoke as a group I carcinogen, ‘carcinogenic to humans’ [3], and it is the most common cause of lung cancer throughout the lifespan [10]. Both firsthand and secondhand tobacco smoke increase the risk of lung cancer, with exposure before the age of 25 leading to higher lifetime lung cancer risk than exposure after age 25 [11,12]. Several studies have shown an increased risk of childhood cancer associated with maternal and/or paternal smoking before conception and/or during pregnancy. A meta-analysis of seven case–control studies found a modest association between maternal smoking during pregnancy and risk of childhood neuroblastoma (OR 1.28; 1.01–1.62), but the data did not support a dose–response relationship [13]. A later meta-analysis of thirteen case–control studies, including seven covered by the earlier analysis, found comparable results for maternal smoking and risk of childhood neuroblastoma (OR 1.22; 1.04–1.44); there was no evidence of a dose–response relationship [7].

An expansive review of the literature found mixed results for exposure to active maternal smoking and passive smoking (including paternal smoking) before or during the index pregnancy; the evidence suggested a slightly increased risk of childhood brain tumors with exposure to active maternal smoking and a modestly increased risk with passive (including paternal) smoking [14]. A pooled analysis of two registry-based studies conducted in France [15] produced a nonsignificant association (OR 1.3; 0.9–1.7) for maternal smoking (ever: never) during pregnancy in relation to neuroblastoma diagnosed before age 6, and there was no evidence of a dose–response relationship and no association with paternal smoking. There was no association between childhood brain tumor and maternal smoking 3 months prior to pregnancy or during any pregnancy trimester. The results were suggestive but inconclusive for any maternal smoking in children with a brain tumor diagnosis before age 5. Paternal smoking 3 months before or during the pregnancy was associated with a modestly increased risk of childhood brain tumor (OR 1.25; 1.03–1.52); although the highest level of smoking was associated with higher risk (OR 1.44;1.13–1.84), an overall dose–response relationship was not evident. The association with paternal smoking was stronger for children with a diagnosis of a brain tumor prior to age five (OR 1.52; 1.14–2.02), and for astrocytoma specifically (OR 1.86; 1.26–2.74) [16]. A meta-analysis based predominately on unpublished data from a study in Greece found no association between maternal smoking during pregnancy and childhood Wilms tumor (14 studies; OR 0.93; 0.80–1.09) [9].

## 5. Diet and Vitamins

There is good evidence of an inverse association between childhood cancer and maternal vitamin supplementation—especially folic acid—during pregnancy. A recent meta-analysis of 17 studies identified reduced risk of childhood leukemia when mothers took folic acid supplementation during pregnancy (OR 0.75; 0.66–0.86) [17]. Subgroup analysis of five of those studies identified a non-significant reduction in risk of AML (OR 0.70; 0.46–1.06), and no significant association with childhood brain tumors [17]. In another meta-analysis of four studies, folic acid intake during pregnancy was associated with a reduced risk of Wilms tumor (OR 0.79; 0.69–0.91) [9]. Prescription iron during pregnancy increased risk for ALL, medulloblastoma and Wilms tumor, but evidence was limited to a single study during the period reviewed, and the authors commented that previous evidence was weak or null [18]. Most developed countries require folic acid fortification of food items such as flour to reduce neural tube defects; this has been mandatory in the United States and Canada since 1998 [19,20]. At a population level, after increases in spina bifida during the early 1990s, a clear downward trend was seen after the mid-1990s. No such trend was seen for pediatric cancers overall, although a decline was seen in Wilms tumor and pancreatic neuroendocrine tumor (PNET). However, there is no evidence to show this was directly related to folic acid food fortification [21].

A case–control study in California showed a reduced risk of ALL in children whose mothers had a higher score on diet quality during the year before pregnancy (OR 0.88; 0.78–0.98). The inverse association was also evident (OR 0.79; 0.68–0.92) for a 5-point increase in diet quality score; no association was seen in children aged 5 or older [22]. Although the findings for AML were similar, they were not statistically significant. Food-specific analyses showed reduced risks of both ALL (OR 0.70; 0.52–0.94) and AML (OR 0.23; 0.08–0.70) in children whose mothers ate fruit at least once daily; although no other single component of the healthy diet was associated with reduced leukemia risk, the authors note a correlation between fruit consumption and overall high quality diet scores [22].

A meta-analysis of two case–control studies showed a lower risk of childhood ALL with maternal consumption of fruits (OR 0.81; 0.67–0.99), vegetables (OR 0.51; 0.28–0.94), and legumes (OR 0.76; 0.62–0.94) “around the time of pregnancy” [23]. In a separate analysis for which pre-pregnancy and pregnancy data were available, inverse associations with ALL were found with folic acid supplementation before pregnancy (only) (OR 0.69; 0.50–0.95) and taking vitamins during pregnancy (OR 0.81; 0.74–0.88) [23]. An inverse association between fish consumption and ALL (OR 0.27; 0.14–0.53) was observed only in younger children (those diagnosed < 5) [23]. A growing area of interest is the role of folic acid in DNA methylation, and epigenetics in childhood cancer etiology [24].

## 6. Caffeine

Women are advised to limit their caffeine consumption during pregnancy [25] and there is now strong evidence of adverse outcomes such as pregnancy loss and low birth weight [26]. For cancer, Milne’s pooled analysis of individual-level data from eight case–control studies confirmed a significantly increased risk of childhood ALL in children of mothers who had consumed two or more cups of coffee daily during pregnancy (OR 1.27; 1.09–1.43) [27]. These findings are built upon two previous meta-analyses that were based on some of the same studies used by Milne, but which used study-level data [28,29]. For example, Cheng’s meta-analysis of seven case–control studies showed a modestly increased risk of acute leukemia with ever coffee drinking during pregnancy overall compared with never/lowest level of coffee drinking (OR 1.22; 1.04–1.43); risk was greater for high-level coffee drinkers (OR 1.72; 1.37–2.16). Similarly, risks of AML (OR 1.35; 1.10–1.66) and ALL (OR 1.26; 1.05–1.50) were modestly elevated when comparing ever coffee drinking with never/lowest coffee drinking, and more elevated for AML (OR 1.58; 1.20–2.08) and ALL (OR 1.65; 1.28–2.12) when comparing the highest level of coffee drinking with never/lowest coffee drinking [28,29]. Another meta-analysis by Thomopoulos et al. assessed twelve case–control studies and found high maternal coffee consumption during pregnancy positively associated with both ALL (OR 1.43; 1.22–1.68) and AML (OR 2.52; 1.59–3.57); dose responses were observed as well [29].

A pooled case–control study from the Childhood Leukemia International Consortium showed a non-significantly increased risk of AML with any coffee consumption during pregnancy compared with none (OR 1.21; 0.92–1.59) and a higher significant risk when more than one cup of coffee daily was compared to none (OR 1.40; 1.03–1.92) [30]. Another case–control study in Australia found no evidence (not statistically significant) of increased risk of brain tumor (OR 1.23; 0.92–1.64) in the children of women who drank any coffee, compared with none, during pregnancy [31]. In children ages 0–4, mothers’ pregnancy consumption of any coffee, compared with none, was associated with increased risk of brain tumor (OR 1.76; 1.09–2.84); the association was stronger for maternal consumption of 2+ cups of coffee per day (OR 2.52; 1.26–5.04), but no association was seen when all ages were combined (OR 1.35; 0.95–2.04).

In general, studies considering risk of coffee consumption in pregnancy and cancer often lack rigorous consideration of biases that might drive reported associations [32]. Confounding factors that hinder analysis include the distinctions between caffeinated and decaffeinated beverages, strength of the beverage, addition of sugar, artificial sweeteners, milk or creamers, concurrent tobacco use, and recall bias. IARC currently designates coffee as “not classifiable as to its carcinogenicity to humans (Group 3)” [33].

## 7. Parental Age

Robust evidence supports an independent association between advanced maternal age and childhood cancer. It is well-established that advanced maternal age is associated with increased risk of chromosomal abnormalities and birth defects in offspring [34,35], which are related to childhood cancer risk [36]. A potential role for epigenetic modification—for example, reduced DNA methylation with increasing maternal age—is being explored as a possible mechanism linking maternal age and childhood cancer [37].

A recent meta-analysis from the Childhood International Leukemia Consortium showed an association between older maternal age (in 5-year increments) and increased childhood ALL in five case–control studies nested in prospective studies (OR 1.05; 1.01–1.08), but not in eleven retrospectively conducted case–control studies [38]. In the same study, older paternal age, also assessed in 5-year increments, was associated with increased risk of childhood ALL both in the nested case–control studies (OR 1.05; 1.01–1.07) and the 11 retrospectively conducted case–control studies (OR 1.04; 1.01–1.07) [38]. In the same report, separate pooled analyses, also assessing 5-year age increments, were performed on fifteen studies for which individual data were available (four nested and eleven retrospective case–control studies). The results showed increasing risk of ALL with increasing paternal age (OR 1.08; 1.04–1.11) but decreasing risk with increasing maternal age (OR 0.92; 0.89–0.96). The associations were more apparent in children with a diagnosis at ages 1–5 years, although this may be due, at least in part, to a substantially higher number of cases and controls in that age group. The authors attempted to separate the influence of maternal and paternal age, but collinearity between paternal and maternal ages impeded these analyses. It will be important to assess these influences independently in the future because there are biological rationales for both as risk factors for childhood ALL.

In a multi-state case–control study, maternal age of 40 or older was associated with increased risk of AML (RR 4.80; 1.80–12.76), and paternal age < 20 was also associated with increased risk of ALL (OR 3.69; 1.62–8.41) [39]. A meta-analysis confirmed the very strong association between AML and advanced maternal age ≥ 40 with a pooled odds ratio of 6.87; 2.12–22.25, for diagnosis before age 1, but not in children of other ages [40]. A review noted a higher risk of brain cancer with higher maternal age, even after adjustment for paternal age [41]. Weak ecological evidence was provided by Kehm’s report that county-level maternal age was significantly associated with a higher annual percent change in incidence of childhood cancer [42].

## 8. Maternal Diabetes

Diabetes mellitus has been widely recognized as a strong risk factor for several cancers in adults, but the role of maternal diabetes in childhood cancers is difficult to disentangle from related maternal factors, such as obesity and pregnancy loss, and offspring factors such as preterm birth and high birth weight (macrosomia), all of which increase the risk of childhood cancer. Nevertheless, there seems to be consensus that maternal diabetes carries increased risk of childhood cancer, especially leukemia, although the relative importance of pre-existing vs. gestational diabetes remains uncertain, and some studies suggest a decreased risk of brain tumor.

A registry linkage study in Finland showed increased risk of childhood cancer in offspring born to mothers with gestational diabetes (OR 1.31; 1.11–1.54) but not with pre-existing maternal diabetes (OR 1.11; 0.73–1.69) [43]. In contrast, in a meta-analysis, two studies showed an association between overall childhood cancer risk and pre-existing (OR 1.41; 1.17–1.70) but not gestational diabetes (OR 1.10; 0.94–1.28); an additional three studies produced positive findings between any maternal diabetes and overall childhood cancer risk, and two studies suggested positive associations that were not statistically significant [44]. The same meta-analysis found associations between maternal diabetes and leukemia (six studies, OR 1.30; 1.15–1.48) and ALL (OR 1.44; 1.27–1.64); the association with ALL was evident for both pre-existing and gestational diabetes. An association with neuroblastoma was of marginal statistical significance (three studies, OR 1.53; 1.00–2.35), and an inverse association was found between maternal diabetes and brain tumors (OR 0.59; 0.39–0.91). Maternal diabetes was not associated with retinoblastoma or lymphoma [44]. A cohort and registry-based study in Sweden, in which Type 1, Type 2, and gestational diabetes were combined, also found a decreased risk of brain tumor (aIRR 0.56; 0.35–0.91) [45]. The same study did not find a statistically significant association between maternal diabetes and risk of childhood cancer overall, but outcome-specific analyses showed an increased risk of childhood leukemia (aIRR 1.47; 1.13–1.92) and ALL (aIRR 1.64; 1.23–2.18) [45].

In analyses of diabetes subtypes, the IRR for gestational diabetes and type 1 diabetes on risk of leukemia, ALL, and brain tumor resembled the effect of combined diabetes on each of these outcomes. A population-based case–control study in California showed an increased risk of any leukemia (OR 1.23; 1.01–1.49) and ALL (OR 1.37; 1.11–1.69) in relation to maternal pre-existing diabetes, but gestational diabetes was not statistically significantly associated with cancer risk [46]. A cohort study in Italy did not find a significant association between pre-existing diabetes and ALL; although the HR was elevated, the confidence interval was wide and included the null (HR 2.7; 0.7–11.1) [47].

## 9. Maternal Obesity

There is mixed evidence of an association between maternal obesity and the risk of childhood cancer. A US study linking birth records and cancer registry data found that maternal obesity (body mass index (BMI) ≥ 40) compared with a normal BMI (18.5–24.9), was associated with a higher risk of any cancer in the offspring (HR 1.32; 1.08–1.62) and specifically of ALL (HR 1.57; 1.12–2.20), but information on maternal diabetes was not available [48]. A population-based case–control study in California provided little evidence of a higher risk of ALL with maternal pre-pregnancy overweight status; an association was seen only with BMI 25-<30 and not with higher BMI levels (30+) [46]. A retrospective cohort study showed a significantly increased risk of childhood cancer in the offspring of women who were obese before they became pregnant but did not distinguish the potentially independent effects of obesity, pre-pregnancy diabetes, and gestational diabetes; in addition, the main result cited in the abstract conflicted with results in the main manuscript so the magnitude of the effect is unclear [49].

## 10. Birth Characteristics and Obstetric History

Several birth characteristics have been shown to increase the risk of certain childhood cancers. In epidemiologic studies, it may be difficult to distinguish the independent impacts on cancer risk of inter-related factors such as low birth weight, preterm birth, multiple gestation, birth order, parental age, birth conditions, infections, smoking, drug use, stress, physical trauma, Cesarean delivery or operative vaginal delivery, and treatments received. Studies are reported below in which specific exposures are investigated.

### 10.1. Birth Weight

High birth weight (>4000 g) is associated with greater risk of several childhood cancers, but with lower risk of others. Either low or high birth weight can be associated with childhood cancers.

There is good evidence that high birth weight is associated with higher risk of childhood leukemias and primary brain tumors [50,51,52,53], but the known association between macrosomia and maternal diabetes was not always considered, limiting the ability to isolate an influence of high birth weight [54]. Building on considerable earlier evidence, O’Neill’s large case–control study based on UK and US cancer registry data found linear increases in total cancer risk per 0.5 kg increase in birth weight in the US (OR 1.06; 1.04–1.08) and UK (OR 1.06; 1.05–1.08) [55]. Statistically significantly higher risks were seen with higher birth weight for several cancers, including leukemia, and tumors of the CNS, kidney, and soft tissue. However, higher birth weight was associated with lower risk of hepatic tumors (US: 0.77; 0.69–0.85; UK: 0.79; 0.71–0.89). Other case–control studies also support the association between high birth weight and ALL in children 5 and under [56,57].

A large population-based cohort study conducted in Australia showed an increased risk of overall childhood cancer diagnoses related to high birth weight (>4 kg; OR 1.4; 1.2–1.6) [58], and a case–control study in Switzerland reported similar results for childhood ALL after adjustment for multiple factors (OR 1.50; 1.19–1.87) [57]. A nationwide case–control study (203 cases) in Greece found no association between CNS tumors and increasing birth weight (assessed in 500 g increments) [8]. A meta-analysis of 41 studies demonstrated an association between higher birth weight (>4000 g vs. ≤4000 g) and childhood CNS tumors overall (OR 1.14; 1.08–1.20) [53]. Subtype analyses demonstrated modest but statistically significant increased risks of low-grade astrocytoma and embryonal tumors, and a moderately elevated risk of high-grade astrocytoma (OR 1.60; 1.21–2.11) with higher birth weight. Significant associations were not found for other tumor types, including ependymoma [53]. A meta-analysis found associations between high birth weight and astrocytoma (n = 11 studies OR 1.60; 1.23–2.09), but heterogeneity was high among studies [50]. A pooled analysis of two population-based, case–control studies in France [59] showed modestly increased risks of neuroblastoma in children age <15 with low (OR 1.4; 1.0–2.0) or high (OR 1.5; 1.1–2.2) birth weight for gestational age. Additionally, a case–control study in the US showed that low (OR 4.46; 1.41–14.1) and high (OR 2.41; 1.09–5.35) birth weight were associated with greater risk of alveolar rhabdomyosarcoma (66 cases); although the results suggested a possible association with embryonal rhabdomyosarcoma (215 cases), the confidence intervals were wide [60].

Low birth weight (<2500 g) is challenging to study because of its close correlation with preterm birth and related risks. The strongest association with cancer is with very low birth weight (<1000 g) which only occurs with very preterm births; Spector et al. note that rather than being causative, very low birth weight “likely signals the involvement of correlated factors” [61]. A meta-analysis of 12 studies showed an association between low birth weight and increased risk of the combined outcome of medulloblastoma and PNET (OR 1.19; 1.02–1.39) but not with astrocytoma or ependymoma [50].

### 10.2. Gestational Age

Associations between gestational age and childhood cancer vary by type of cancer. A meta-analysis of five studies showed no association between increased risk of primary CNS tumors and preterm birth, compared with birth at 37+ weeks [62]. Analysis of another five studies also found no association between preterm birth and neuroblastoma [62]. A meta-analysis of seven studies demonstrated an increased risk of AML (OR 1.42; 1.21–1.67) but not ALL in children born preterm (<37 weeks) [63]. A meta-analysis of six studies produced a borderline association between preterm birth and increased AML risk (OR 1.20; 1.00, 1.44) [64]. A study in Australia showed increased risk of hepatic tumors related to extreme preterm (<28 weeks) birth (aOR = 12.7; 3.3–48.3) but did not adjust for prenatal radiation exposures from medical imaging, which may increase risk of hepatic tumor [58]. A population-based record linkage study in Sweden found an association between preterm birth and rhabdomyosarcoma [65]. In a meta-analysis of six studies, a borderline association was observed between post-term birth (i.e., after 42 weeks) and risk of AML (OR 1.20; 1.00–1.43); a subgroup analysis of two cohort studies indicated an association between post-term birth and ALL (OR 1.21; 1.06, 1.39) [64].

### 10.3. Phototherapy

Phototherapy to reduce bilirubin levels in neonates is generally administered using narrow-spectrum LED blue light in the wavelength 475 nm, but there is no standard required in clinical practice [66]. Two studies have indicated a 2-fold or greater increased risk of cancer in children who underwent phototherapy for neonatal jaundice [67,68]. However, another study reported that adjustment for confounding variables such as bilirubin, congenital anomalies and chromosomal disorders attenuated most of the associations such as the original findings of increased risk of leukemia and liver cancer [69].

### 10.4. Multiple Gestation

A large registry-based case–control study in Switzerland (1403 cases) found an increased overall risk of childhood leukemia associated with multiple gestations compared with single births after adjustment for other factors (OR 1.89, 1.24–2.86); the association was similar for ALL [57]. However, that result contradicts the findings of studies outside of our study period.

### 10.5. Birth Order

The evidence of an association between birth order and childhood cancer is mixed. A US-based case–control study (Children’s Oncology Group) of rhabdomyosarcoma found no evidence of an association with birth order when comparing second- and third-born children with firstborns [60]. A pooled analysis based on six cohorts from the International Childhood Cancer Cohort Consortium showed a lower risk of childhood leukemia (185 cases) with increasing birth order (HR 0.88; 0.77–0.99), and specifically of ALL (136 cases) (HR 0.85; 0.73–0.99) [70]. The same study provided evidence that paternal age and birth weight may modify the association between being later-born and leukemia; children born at <3 kg with fathers aged 35+ had the lowest risk (HR 0.18, 0.06–0.50) [70]. Yan’s meta-analysis reported an 8% higher risk of ALL among firstborns for ALL (OR 1.08; 1.00–1.16) [71].

A case–control study in Greece of brain tumors affecting children ages 0–14 (203 cases, 406 controls) showed that, in comparison with firstborn children, second-born children had a reduced risk of brain tumors (aOR 0.60; 0.40–0.89); risk was further reduced in the third-born children (aOR 0.34; 0.18–0.63) [8]. The results were similar for astrocytomas, but not for embryonal tumors. A meta-analysis of 16 case–control studies (32,439 cases) showed second-borns, compared with firstborns, had a small yet significantly increased risk of brain tumors (OR 1.04; 1.01–1.07). A reduction in brain tumor risk was seen for fourth-born children compared with firstborns (OR 0.85; 0.78–0.92) in the analysis of case–control studies, but not in the analysis of three cohort studies (4515 incident cases), when comparing later born to first born children (HR 1.00, 0.96–1.05) [72]. It is unclear whether later birth order relates to biological factors or other factors within larger families, such as infectious disease risk and parental age.

### 10.6. Cesarean Section and Operative Vaginal Delivery

The evidence available for the impact of mode of delivery on childhood cancer risk is mixed. Cesarean delivery alters the conditions in which the baby experiences early life, including colonization with vaginal bacteria, the physiological changes including stress that occur during labor, and recovery by the mother, which can affect breast-feeding. The indication for cesarean delivery must also be considered when interpreting any potential association between cesarean delivery and childhood cancer, and there must be appropriate adjustment for confounding variables such as gestational age, birth weight, birth order and plurality, maternal age, and any birth conditions that led to cesarean delivery. Subsequent individual studies, including a population-based data linkage study in Australia, identified excess cancer risk associated with cesarean delivery (aOR 1.2; 1.1–1.4) after adjustment for preterm birth and birth weight ≥ 4 kg. The same study showed that neonatal treatment with nitric oxide for respiratory failure was independently associated with an increased risk of childhood cancer (aOR 8.6; 4.3–17.4) [58]. The case–control study in Greece by Georgakis et al. noted above in the section on birth order, identified lower risk of childhood brain tumors (aOR 0.67; 0.45–0.99) in children born by cesarean section, and a higher risk among children born by operative vaginal deliveries including forceps and vacuum assisted vaginal deliveries (aOR 7.82; 2.18–28.03) [8].

## 11. Assistive Reproductive Technologies (ARTs)

At present, there is no evidence that children conceived through ART have increased risk of childhood cancer. ARTs are in vitro interventions on human oocytes, sperm or embryos for reproduction [73]. Two large meta-analyses, covering 24 case–control studies and 11 cohort studies, showed no increased risk of childhood cancers with assistive reproductive technologies or in vitro fertilization [74,75].

## 12. Prenatal Exposure to Medications Used During Pregnancy

### 12.1. Diethylstilbestrol (DES)

DES is an established human transplacental carcinogen that was given to pregnant women to prevent pregnancy losses. In 1971, about three decades after DES was introduced, a small case–control study showed a strong association between prenatal DES exposure and clear cell adenocarcinoma (CCA) of the cervix/vagina in girls and young women [76]. A later prospective study estimated a 40-fold greater incidence of CCA in the prenatally DES-exposed females [77]. A meta-analysis supported a long-suspected association with testicular cancer in prenatally DES-exposed young men [78].

A small prospective study suggested a possible increased risk of ovarian cancer in the granddaughters of women given DES during pregnancy [79]. Case reports involving granddaughters of pregnancy-exposed women have described an ovarian cancer occurring in a 15 year-old [80], and CCA in an 8-year-old [81]. If confirmed, intergenerational effects might be due to epigenetic alterations that contribute to cancer susceptibility.

### 12.2. Other Hormones

Evidence for an association between childhood cancers and maternal use of hormones other than DES is weak due to limited research. In a large retrospective cohort study conducted in Denmark, development of any leukemia was associated with use of hormonal contraception within 3 months before conception (HR 1.46; 1.09–1.96); no significant association was seen with hormone use after conception (HR 1.78; 0.95–3.31). For non-lymphoid leukemia, the hazard ratio for use prior to conception was 2.17 (1.22–3.87) and 3.87 (1.48–10.15) for use after conception. The authors estimated an attributable risk of 1 case of leukemia per 50,000 exposed children [82].

### 12.3. Antibiotics and Antiretrovirals

There is mixed evidence of an association between maternal use of antibiotics during pregnancy and increased risk of cancer in the offspring. Examining this association is complicated by the challenge of disentangling the adverse effects of prenatal exposure to maternal antibiotics from that of prenatal exposure to maternal infection, which has an established biological precedent (e.g., health consequences of prenatal maternal infection with rubella virus [83]). A retrospective cohort study showed no overall association between childhood cancer and prenatal exposure to maternal antibiotics during pregnancy, but increased risk of cancer (HR 1.5; 1.1–1.9) was seen for maternal antibiotic use during the first trimester [84]. A registry-based study in Denmark did not find an association between childhood leukemia and prenatal exposure to maternal use of antibiotics [85]. However, a registry-based prospective study of childhood cancer in Denmark and Sweden found associations with cancer (all types combined) in very young children (age < 5); the HRs were 1.15 (1.02–1.31) for any prenatal antibiotic exposure; 1.37 (1.09–1.72) for exposure during the third trimester; and 1.29 (1.07–1.55) for multiple prenatal exposures [86]. Analyses of specific cancer types showed modest and borderline associations with AML and ALL before age 5. In addition, a record-based, case–control study in the UK found associations between AML and prenatal exposure to antibacterial/penicillin (OR 1.58; 1.05–2.38), and between sarcoma and prenatal exposure to systemic antibacterials (OR 1.80, 1.03–3.16) [18].

Evidence is mixed for the relationship between prenatal exposure to antiretrovirals and childhood cancer risk. A review did not indicate an association between children’s cancer risk and maternal pregnancy treatment with antiretroviral medications aimed at preventing perinatal HIV transmission [87]. However, a study in France showed that prenatal exposure to maternal antiretrovirals was associated with increased cancer risk in childhood; the association was strongest for children born preterm (<33 weeks), and for those exposed prenatally to didanosine (prescribed to prevent perinatal HIV transmission) alone or in combination with other antiretrovirals. Although the association with didanosine combined with other antiretrovirals was not of statistical significance (HR 2.9; 0.9–9.3), a strong association was observed for prenatal exposure to didanosine (with or without other retrovirals) during the first trimester (HR 5.5; 2.1–14.4). This finding was based on seven cases, only one of which was exposed after the first trimester, precluding an analysis of later exposure effects. No associations were observed for several other antiretroviral agents studied [88].

### 12.4. Antipyretics/Analgesics

A hospital-based case–control study in Brazil assessed risk of ALL and AML in young children (age < 24 months) whose mothers took acetaminophen during pregnancy [89]. The findings suggested a strongly reduced risk of childhood leukemia for maternal acetaminophen use at any time during pregnancy, but the inverse association with ALL was statistically significant only with use during the first (OR 0.39; 0.17–0.93) or second trimester (OR 0.37; 0.16–0.88). For AML, results were statistically significant only for second trimester use (OR 0.11; 0.02–0.97) [89]. The same study in Brazil found an increased risk of ALL in children < 24 months of age with mothers’ preconception use of the NSAID dipyrone/metamizole (OR 1.63; 1.06–2.53); in analyses confined to children < 12 months of age, ALL risk appeared to be doubled with exposure during the first two trimesters, but small numbers prohibited confounder adjustment. In these studies, it is important to consider the underlying condition that led to acetaminophen use.

There were no significant associations between dipyrone and AML. Dipyrone was banned in the US and in most westernized countries several decades ago but continues to be used in Latin America [89].

### 12.5. Other Medications and Drug Use

Miscellaneous other medication exposures have not been extensively explored and need further assessment. Borderline increased risk of ALL in prenatally exposed offspring was previously seen with prenatal maternal antihistamine use, maternal or paternal use of mind-altering drugs, and paternal use of amphetamines or diet pills [90]. More recently, a population-based cohort study using national registers in Denmark identified no excess risk of childhood cancer associated with prenatal exposure to the mother’s antidepressant use [91].

## 13. Medical Ionizing Radiation During Pregnancy

The link between childhood (postnatal) ionizing radiation and malignancy is well-established (reviewed in Parts 1 [1] and 3). In contrast, most studies of prenatal radiation exposure due to diagnostic imaging of the pregnant mother have not found an association with childhood cancer. It is generally agreed there is no safe level of radiation exposure [92,93], but studies of prenatal exposure to low doses of diagnostic radiation (e.g., diagnostic X-ray) may lack sufficient statistical power to detect small increases in cancer risk in prenatally exposed children. A meta-analysis of case–control studies conducted since 1970 assessed prenatal exposure to ionizing radiation due to diagnostic medical imaging and found no association with overall cancer (no effect measure given), leukemia (OR 1.08; 0.90–1.28; 4 studies) or brain tumor (OR 0.93; 0.68–1.28; 2 studies) [94]. Most of the included studies assessed cancers occurring at ages 0–16; one of the two studies included in the brain tumor analysis assessed diagnoses in children ages 7–19. The authors note that reductions in radiation dose over time probably account for the positive associations noted by earlier studies and the null findings in more recent studies.

Studies not included in the meta-analysis by Abalo also found no association between childhood cancer and pregnant mothers’ exposure to diagnostic medical radiation. Results from a case–control study based on the international MOBI-Kids study did not find statistically significant associations between prenatal estimated radiation exposure and brain tumors [95]. Although the OR increased with higher estimated doses, none approached statistical significance. It should be noted, however, that most studies assessed exposure to diagnostic X-ray; dose levels are substantially higher with computed tomography (CT scans) and even higher for many interventional radiology procedures. Occupational exposure to radiation during pregnancy is addressed in Part 3.

## 14. Limitations

This review summarizes evidence from English-language papers published from 2014 to early 2021. Evidence published before or after our time frame may be different than the evidence presented here; this point is especially true for multiple gestation, because earlier and more recent studies show a decreased cancer risk, whereas the single study we cite shows increased risk [57]. We encourage future efforts to synthesize this past assessment with more recent literature to update our findings. We also note the potential for bias against inclusion of research from non-English-speaking nations. More generally, etiologic studies of pediatric cancer are limited by exposure recall and measurement, and small sample sizes when cancer subtypes are uncommon. For pregnancy-related factors, the interconnectedness of many prenatal risk factors makes it difficult to tell which act independently rather than as confounders or surrogates for something else.

## 15. Conclusions

Several of the risk exposures discussed in this series, such as smoking, alcohol, and coffee consumption during pregnancy, could be readily avoided by individuals who are considering pregnancy, with potential health benefits for both them and their offspring. Additionally, there is strong evidence for cancer prevention strategies including healthy nutrition during pregnancy. Some of this information is either too recent or tentative to be considered for inclusion in clinical guidelines and is intended to guide informed decision making between parents and their clinicians.

In any case, there is convincing evidence for a need for prenatal education to improve maternal and child health, and these efforts may also reduce the chance of developing pediatric cancer.

## Figures and Tables

**Table 1:** Content in each of the three parts of this review.

Part 1 [1] Child Factors	Part 2 Parental Pre-Pregnancy and Pregnancy Factors	Part 3 Environmental and Occupational Factors
Genetic predisposition	Alcohol	Air Quality - Outdoor exposures - Indoor exposures
Birth defects	Smoking	Radiation exposures - Outdoor sources - Ionizing radiation - Non-ionizing radiation
Prior cancer and associated treatments	Diet and vitamins	Occupational exposures:
Medical ionizing radiation	Caffeine	- Benzene
Ultraviolet (UV) light	Parental age	- Miscellaneous other
Organ transplantation	Maternal diabetes	- Agricultural animals
Medications in childhood	Maternal obesity	- Agricultural pesticides
Diet and breastfeeding	Birth characteristics and obstetric history	
Body mass index	- Birth weight	
Infections	- Gestational age	
Vaccinations	- Phototherapy for jaundice	
Allergies	- Multiple gestation	
	- Birth order	
	- Cesarean section and instrumental delivery	
	Assistive reproductive technologies	
	Medications during pregnancy	
	- Diethylstilbestrol (DES)	
	- Other Hormones	
	- Antibiotics and antiretrovirals	
	- Antipyretics/analgesics	
	- Other medications and drug use	
	Medical ionizing radiation during pregnancy	

**Table 2:** Summary of the strength of evidence for each pregnancy-related factor with childhood cancer *.

Exposure	Notes
Strong evidence of association with childhood cancer	
Alcohol use during pregnancy	Maternal consumption of alcohol, an International Agency for Research on Cancer (IARC) [3] group I carcinogen, is associated with increased risk of several childhood cancers.
Cigarette smoking during pregnancy	Tobacco smoke, an IARC group I carcinogen [3], is associated with higher risk of several childhood cancers; maternal and paternal smoking are implicated, both before conception and during pregnancy.
Diet and vitamins	Lower risk of certain childhood cancers is seen with folic acid supplementation and consumption of fruits, vegetables, and legumes during pregnancy.
Caffeine	IARC describes coffee as “not classifiable as to its carcinogenicity to humans” [3] but there is growing evidence of a link between coffee consumption during pregnancy and increased risk of childhood leukemia in the offspring.
Maternal diabetes	Pre-existing maternal diabetes is associated with an overall increased risk of cancer and leukemia, and with acute lymphoblastic leukemia (ALL), but evidence is mixed regarding the role of gestational diabetes.
Gestational age	There is evidence that preterm birth increases the risk of several cancers. Post-term birth may increase risk of leukemia overall.
High birth weight	There is good evidence that high birth weight increases overall cancer risk and risk of leukemia, CNS cancers, and several other malignancies.
Low birth weight	Evidence indicates that low birth weight is associated with increased risk of alveolar rhabdomyosarcoma, primitive neuroectodermal tumor (PNET), and medulloblastoma. Both high and low birth weight may be related to an increased risk of neuroblastoma.
Diethylstilbestrol	IARC classifies diethylstilbestrol (DES) as a group 1 carcinogen [3]. In utero exposure to DES is causally related to a strong increased risk of cervical/vaginal clear cell adenocarcinoma in girls and young women.
Parental age	Older parental age is associated with increased risk of childhood ALL; collinearity affects the separate analysis of maternal and paternal age.
Mixed evidence of association with childhood cancer	
Maternal obesity	There is mixed evidence of an association with childhood cancer, independent of maternal diabetes and the child’s birth weight.
Birth order	Later birth order may be related to decreased leukemia risk, but there is mixed evidence for other cancer types, including brain tumors.
Cesarean/instrumental delivery	There is mixed evidence that C-section increases total cancer risk in offspring. The risk of brain tumor may be lower in C-section delivery but higher in instrumental vaginal delivery.
Radiation during pregnancy	Recent studies provide mixed evidence of a link between childhood cancer and exposure to radiation in utero, perhaps in part because of the establishment of ionizing radiation as a class I carcinogen [4,5]; this has led to substantially reduced use of medical imaging during pregnancy, affecting the ability to study lower dose or less frequent exposure.
Weak or no evidence of association with childhood cancer	
Phototherapy for neonatal jaundice	After adjustment for potential confounders including the causes of neonatal jaundice, little evidence was found for an association with childhood cancer.
Multiple gestation	A possible association has been found in a single study comparing multiparity to single birth outcomes with leukemia, but more evidence is needed.
Assisted reproductive therapies (ARTs)	Studies have not shown an association between ART and childhood cancer risk.
Other hormones (excluding DES)	Weak evidence due to limited research; one study has shown an association between pregnancy use of non-DES hormones and increased cancer in the offspring consequently.
Antibiotics/antiretrovirals	Evidence of increased cancer in children exposed in utero to antibiotics is weak. A possible link with cancer has been reported for in utero exposure to antiretrovirals.
Analgesics/Antipyretics	Weak evidence due to limited research; one study suggests reduced risk of ALL with acetaminophen use during first or second trimester, and of acute myeloid leukemia (AML) during third trimester. Prenatal exposure to Dipyrone may increase risk of ALL in children < 4 months.
Other medications	Evidence for most specific medications is weak due to limited research; studies of iron taken during pregnancy have produced inconsistent results.

* The evidence of an association is considered strong if a clear majority of studies indicate the exposure is linked to at least one type of cancer. The same exposure may be related inconsistently or weakly, or unrelated to other types of cancer.

## Data Availability

The search strategy is provided by Ricci et al. [1], which allows the reader to reproduce the search that identified papers for this paper.
